# A Perspective on the Comparative Antileukemic Activity of 5-Aza-2′-deoxycytidine (Decitabine) and 5-Azacytidine (Vidaza)

**DOI:** 10.3390/ph5080875

**Published:** 2012-08-21

**Authors:** Richard L. Momparler

**Affiliations:** Département de Pharmacologie, Service d’ Hématologie et Oncologie, Centre de Recherche, CHU-Saint-Justine, Université de Montréal, Montréal, Québec H3T 1C5, Canada; Email: richard.l.momparler@umontreal.ca; Tel.: +1-514-345-4931 (ext 6140); Fax: +1-514-345-4801

**Keywords:** 5-aza-2′-deoxycytidine, 5-azacytidine, leukemia, chemotherapy, DNA methylation

## Abstract

5-Aza-2′-deoxycytidine (5-AZA-CdR, decitabine, Dacogen®) and 5-azacytidine (5-AC, Vidaza®) are epigenetic agents that have been approved for the clinical treatment of the hematological malignancy myelodysplastic syndrome (MDS) and are currently under clinical evaluation for the treatment of acute myeloid leukemia (AML). Most investigators currently classify 5-AZA-CdR and 5-AC as inhibitors of DNA methylation, which can reactivate tumor suppressor genes silenced by this epigenetic event. Examination of the pharmacology of these analogues reveals important differences with respect to their molecular mechanism of action. The action of 5-AZA-CdR is due to its incorporation into DNA. 5-AC is a riboside analogue that is incorporated primarily into RNA. A small fraction of 5-AC is converted to its deoxyribose form by ribonucleotide reductase and subsequently incorporated into DNA. The incorporation of 5-AC into RNA can interfere with the biological function of RNA and result in an inhibition protein synthesis. Microarray analysis revealed that both these analogues target the expression of different cohorts of genes. Preclinical studies show that 5-AZA-CdR is a more effective antileukemic agent than 5-AC. One explanation for this observation is that 5-AC blocks the progression of some leukemic cells from G_1_ into S phase, and this protects these cells from the chemotherapeutic action of this riboside analogue related to its incorporation into DNA. However, differences in chemotherapeutic efficacy of these related analogues have not been clearly demonstrated in clinical trials in patients with hematological malignancies. These observations should be taken into consideration in the design of new clinical trials using 5-AZA-CdR or 5-AC in patients with MDS and AML.

## 1. Introduction

Aberrant DNA methylation is an epigenetic event that can play a key role in the etiology of cancer [1]. Many genes that suppress leukemogenesis are silenced by DNA methylation for both acute lymphoid leukemia (ALL) and acute myeloid leukemia (AML) [2,3]. Since epigenetic modifications are reversible, they are interesting targets for chemotherapeutic intervention. Preclinical studies have shown that both 5-aza-2′-deoxycytidine (5-AZA-CdR, decitabine, Dacogen®) and 5-azacytidine (5-AC, Vidaza®) are potent antileukemic agents [4,5]. Clinical trials on patients with hematological malignancies have lead to the approval of 5-AZA-CdR and 5-AC for the therapy of myelodysplastic syndrome (MDS) [6,7,8,9]. Both these nucleoside analogues show efficacy against AML [10,11,12,13], but have not been yet approved for its treatment. Currently, there are several clinical trials on 5-AZA-CdR and 5-AC in combination with histone deacetylase inhibitors in patients with cancer [14,15,16]. Many clinical investigators that use 5-AZA-CdR or 5-AC consider these agents prototypes inhibitors of DNA methylation. However, careful analysis of the preclinical studies of these analogues indicates that there are important differences in their molecular actions. This review will discuss these differences and their relevance to clinical therapy in patients with leukemia.

## 2. Metabolism

**Figure 1:**
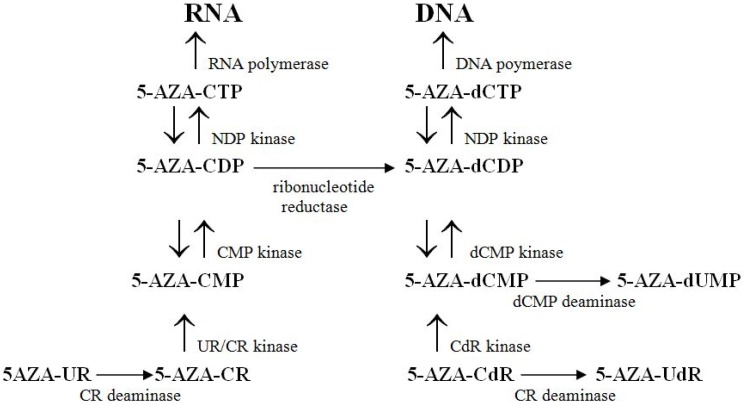
Metabolism of 5-aza-2′-deoxycytidine (5-AZA-CdR) and 5-azacytidine (5-AZA-CR). Both these nucleoside analogues are pro-drugs that have to be activated by phosphorylation. Deoxycytidine kinase activates 5-AZA-CdR, which is converted to its triphosphate form by kinases and incorporated into DNA. Cytidine (CR) deaminase inactivates both 5-AZA-CdR and 5-AZA-CR. dCMP deaminase inactivates 5-AZA-CdR monophosphate (5-AZA-dCMP). 5-AZA-CR is activated by uridine/cytidine (UR/CR) kinase, phosphorylated to its triphosphate and incorporated into RNA. Ribonucleotide reductase converts 10–20% of the diphosphate form of 5-AZA-CR (5-AZA-CDP)) to its deoxyribose form (5-AZA-dCDP), which is phosphorylated to 5-AZA-dCTP and incorporated into DNA.

The metabolism of 5-AZA-CdR and 5-AC is summarized in Figure 1. 5-AZA-CdR and 5-AC are prodrugs that are activated by phosphorylation by deoxycytidine kinase and uridine/cytidine kinase, respectively. Leukemic cells lacking deoxycytidine kinase are drug resistant to 5-AZA-CdR [17]. CR deaminase inactivates both 5-AZA-CdR and 5-AC by deamination. After conversion to its triphosphate, 5-AZA-dCTP is rapidly incorporated into DNA. About 80–90% of 5-AC after conversion to it triphosphate, 5-AZA-CTP is incorporated into RNA; after its conversion to deoxyribose form by ribonucleotide reductase about 10–20% of 5-AC is incorporated into DNA [18,19].

## 3. Pharmacological Action

The incorporation of 5-AZA-CdR and 5-AC into DNA is responsible for their inhibition of DNA methylation. The demethylation of DNA by these analogues leads to the reactivation of tumor suppressor genes that were silenced by aberrant DNA methylation [1]. 5-AZA-CdR is a more potent inhibitor of DNA methylation and proliferation of leukemic cells than 5-AC [18,20,21,22].

Preclinical studies indicate that the incorporation of 5-AC into RNA also contributes to its antineoplastic activity. It was reported that the incorporation of 5-AC into RNA reduced the activity of tRNA in protein synthesis [23]. The presence of 5-AC in RNA interferes with its biological function [24]. Several groups have reported that 5-AC inhibits protein synthesis [18,25]. Recently, 5-AC, but not 5-AZA-CdR, was shown to inhibit cytosine methylation in tRNA^asp^ [26], which can also modify its function. The effect of 5-AC on RNA function and protein synthesis was most likely responsible for the liver and kidney toxicity observed in clinical studies [27,28]. These reports suggest that the antineoplastic action of 5-AC is due to both its incorporation into RNA and DNA. This is probably the major reason for the differences between 5-AZA-CdR and 5-AC with respect to changes in global gene expression, as shown by microarray analysis [18,20].

## 4. Relationship between Mode of Action and Antileukemic Activity

The key question is which of these nucleoside analogues, 5-AZA-CdR or 5-AC, has the greatest chemotherapeutic potential in the treatment of leukemia. In this regard, it should be noted that due to its effect on RNA function and protein synthesis, 5-AC has the potential to interfere with cell cycle transit, since the progression of cells from G_1_ to S phase is dependent on these molecular events [29]. 5-AC has also been shown to inhibit the progression of cells from G_1_ into S phase [30]. The inhibition of the progression of some leukemic cells into S phase by 5-AC has the potential to limits its antineoplastic action, because if some cells in G_1_ phase are blocked from entering S phase, 5-AC cannot inhibit DNA methylation in these cells. This latter event can permit some cells to escape from part of its therapeutic activity. In this regard, 5-AZA-CdR does not inhibit the progression of G_1_ phase cells into S phase [31,32].

Preclinical studies on the relative antileukemic activity of 5-AZA-CdR and 5-AC provide some insight of the importance of the action of 5-AC on cell cycle progression. In a clonogenic assay 5-AZA-CdR was about 10-fold more potent than 5-AC on L1210 leukemic cells [22]. In addition, 5-AZA-CdR produced a much greater inhibition of DNA methylation than 5-AC on these leukemic cells. The mouse model of L1210 leukemia was used to compare the *in vivo* antineoplastic action of these two analogues. A summary of these data is shown in Table 1 [22].

**Table 1:** Comparison of antineoplastic activity of 5AZA-CdR and 5AC in mouse model of L1210 leukemia.

Drug	Dose *	Survival time **	Increase in survival	Cures
5-AC	24.1 mg/kg	13.3 ± 1.1 days	115%	0%
5-AZA-CdR	20.6 mg/kg	48.0 ± 2.5 days	674%	60% ***

* 15 h i.v. infusion; ** Mice received i.v. injection 10^5^ L1210 leukemic cells, control mice survived 6.1 ± 0.5 days; *** Mice survival ≥60 days [22].

The mice were injected i.v. with 10^5^ L1210 leukemic cells and 24 h later administered a 15 h i.v. infusion of 5-AZA-CdR (20.6 mg/kg) or 5-AC (24.1 mg/kg), which increased the life span of the leukemic mice by 674% and 115%, respectively. Remarkably, 5-AZA-CdR cured 60% of the mice, whereas no cures were observed with 5-AC. A cure was defined as mice that survived ≥60 days after i.v. injection of leukemic cells. In this mouse model the L1210 cells are a prototype of leukemic stem cells since one cell will produce death from leukemia in 14 days [4]. Since the L1210 leukemic cells have a doubling time of about 12 h, all of the cells should have entered the S phase during the 15 h infusion. One explanation for the marked differences in chemotherapeutic activity between these analogues is that the action of 5-AC on RNA and protein function blocks the cell cycle progression of some leukemic cells into S phase, limiting its curative action. It should be noted that in this mouse model of L1210 leukemia the antineoplastic action of 5-AZA-CdR correlates with its inhibition of DNA methylation [33], whereas 5-AC is a very weak inhibitor of DNA methylation [18,22].

## 5. Conclusions

In summary, the incorporation of 5-AC into RNA is responsible for part of its cytotoxic action on cells; it may also limit its own therapeutic activity. Preclinical data indicate that 5-AZA-CdR is a more effective antileukemic agent than 5-AC. The modes of action of these analogues are not identical [34]. Whether this difference in antineoplastic activity between these two cytosine nucleoside analogues will also be observed in the clinical treatment of hematological malignancies can only be determined by randomized clinical trials using the optimal dose schedule for each agent. It is interesting to note that some patients with MDS that show clinical resistance to 5-AC can respond to 5-AZA-CdR therapy [35]. Can 5-AC play an important role in the therapy of hematological malignancies using 5-AZA-CdR? Leukemic cells from patients that are deficient in deoxycytidine kinase are resistant to 5-AZA-CdR [17,36]. Since 5-AC is activated by uridine/cytidine kinase, it should be effective against deoxycytidine kinase-deficient cells. The potential of 5-AC to overcome drug resistance to 5-AZA-CdR can be investigated in a preclinical study using a leukemic cell line deficient in deoxycytidine kinase. The potential of 5-AC to overcome drug resistance to 5-AZA-CdR can be investigated by using a leukemia cell line deficient in deoxycytidine kinase. It is also possible that some leukemic cells may be resistant to the demethylation action of 5-AZA-CdR. The inhibitory action of 5-AC on RNA function and its action on the expression of a different cohort of genes have the potential to eradiate these 5-AZA-CdR-resistant cells [37]. This approach can be also investigated in clinical trials. One of the major problems in the chemotherapy of hematological malignancies is the maintenance of complete remission. Patients with MDS or AML induced into complete remission with low dose 5-AZA-CdR are usually continue on low-dose therapy to maintain the remission. However, drug resistance can develop during repetitive low-dose treatments. One approach to overcome this problem is to alternate the maintenance therapy. For example, for every two to three cycles of 5-AZA-CdR, one can administer a single cycle of 5-AC. This approach merits investigation for the treatment of hematological malignancies.

## References

[1] Esteller M. (2008). Epigenetics in cancer. N. Engl. J. Med..

[2] Davidsson J., Lilljebjörn H., Andersson A., Veerla S., Heldrup J., Behrendtz M., Fioretos T., Johansson B. (2009). The DNA methylome of pediatric acute lymphoblastic leukemia. Hum. Mol. Genet..

[3] Deneberg S., Grövdal M., Karimi M., Jansson M., Nahi H., Corbacioglu A., Gaidzik V., Döhner K., Paul C., Ekström T.J., Hellström-Lindberg E., Lehmann S., Johansson B. (2010). Gene-specific and global methylation patterns predict outcome in patients with acute myeloid leukemia. Leukemia.

[4] Momparler R.L., Gonzales F.A. (1978). Effect of intravenous infusions of 5-Aza-2′-deoxycytidine on survival time of mice with L1210 leukemia. Cancer Res..

[5] Presant C.A., Vietti T., Valeriote F. (1975). Kinetics of both leukemic and normal cell population reduction following 5-azacytidine. Cancer Res..

[6] Rüter B., Wijermans P.W., Lübbert M. (2006). Superiority of prolonged low-dose azanucleoside administration? Results of 5-aza-2′-deoxycytidine retreatment in high-risk myelodysplasia patients. Cancer.

[7] Issa J.P., Garcia-Manero G., Giles F.J., Mannari R., Thomas D., Faderl S., Bayar E., Lyons J., Rosenfeld C.S., Cortes J., Kantarjian H.M. (2003). Phase 1 study of low-dose prolonged exposure schedules of the hypomethylating agent 5-aza-2′-deoxycytidine (decitabine) in hematopoietic malignancies. Blood.

[8] Silverman L.R., Demakos E.P., Peterson B.L., Kornblith A.B., Holland J.C., Odchimar-Reissig R., Stone R.M., Nelson D., Powell B.L., DeCastro C.M., Ellerton J., Larson R.A., Schiffer C.A., Holland J.F. (2002). Randomized controlled trial of azacitidine in patients with the myelodysplastic syndrome: A study of the cancer and leukemia group B. J. Clin. Oncol..

[9] Lübbert M., Suciu S., Baila L., Rüter B.H., Platzbecker U., Giagounidis A., Selleslag D., Labar B., Germing U., Salih H.R. (2011). Low-dose decitabine versus best supportive care in elderly patients with intermediate- or high-risk myelodysplastic syndrome (MDS) ineligible for intensive chemotherapy: Final results of the randomized phase III study of the european organisation for research and treatment of cancer leukemia group and the german MDS study group. J. Clin. Oncol..

[10] Lübbert M., Rüter B.H., Claus R., Schmoor C., Schmid M., Germing U., Kuendgen A., Rethwisch V., Ganser A., Platzbecker U. (2012). A multicenter phase II trial of decitabine as first-line treatment for older patients with acute myeloid leukemia judged unfit for induction chemotherapy. Haematologica.

[11] Kantarjian H.M., Thomas X.G., Dmoszynska A., Wierzbowska A., Mazur G., Mayer J., Gau J.P., Chou W.C., Buckstein R., Cermak J. (2012). Multicenter, randomized, open-label, phase III trial of decitabine versus patient choice, with physician advice, of either supportive care or low-dose cytarabine for the treatment of older patients with newly diagnosed acute myeloid leukemia. J. Clin. Oncol..

[12] Al-Ali H.K., Jaekel N., Junghanss C., Maschmeyer G., Krahl R., Cross M., Hoppe G., Niederwieser D. (2012). Azacitidine in patients with acute myeloid leukemia medically unfit for or resistant to chemotherapy: a multicenter phase I/II study. Leukemia Lymphoma.

[13] Maurillo L., Venditti A., Spagnoli A., Gaidano G., Ferrero D., Oliva E., Lunghi M., D'Arco A.M., Levis A., Pastore D. (2012). Azacitidine for the treatment of patients with acute myeloid leukemia: Report of 82 patients enrolled in an Italian compassionate program. Cancer.

[14] Gore S.D., Baylin S., Sugar E., Carraway H., Miller C.B., Carducci M., Grever M., Galm O., Dauses T., Karp J.E. (2006). Combined DNA methyltransferase and histone deacetylase inhibition in the treatment of myeloid neoplasms. Cancer Res..

[15] ClinicalTrials.gov (Decitabine). http://clinicaltrials.gov/ct2/results?intr="Decitabine".

[16] ClinicalTrials.gov (Vidaza). http://clinicaltrials.gov/ct2/results?intr="Vidaza".

[17] Momparler R.L., Momparler L.F. (1989). Chemotherapy of L1210 and L1210/ARA-C leukemia with 5-Aza-2′-deoxycytidine and 3-deazauridine. Cancer Chemother. Phamacol..

[18] Hollenbach P.W., Nguyen A.N., Brady H., Williams M., Ning Y., Richard N., Krushel L., Aukerman S.L., Heise C., MacBeth K.J. (2008). A comparison of azacitidine and decitabine activities in acute myeloid leukemia cell lines. PLoS One.

[19] Li L.H., Olin E.J., Buskirk H.H., Reineke L.M. (1970). Cytotoxicity and mode of action of 5-azacytidine on L1210 leukemia. Cancer Res..

[20] Flotho C., Claus R., Batz C., Schneider M., Sandrock I., Ihde S., Plass C., Niemeyer C.M., Lübbert M. (2009). The DNA methyltransferase inhibitors azacitidine, decitabine and zebularine exert differential effects on cancer gene expression in acute myeloid leukemia cells. Leukemia.

[21] Qiu X., Hother C., Ralfkiær U.M., Søgaard A., Lu Q., Workman C.T., Liang G., Jones P.A., Grønbæk K. (2010). Equitoxic doses of 5-azacytidine and 5-aza-2′-deoxycytidine induce diverse immediate and overlapping heritable changes in the transcriptome. PLoS One.

[22] Momparler R.L., Momparler L.F., Samson J. (1984). Comparison of the antileukemic acitivity of 5-aza-2′-deoxycytidine, 1-β-D-arabinofuranosylcytosine and 5-azacytidine against L1210 leukemia. Leuk. Res..

[23] Momparler R.L., Siegel S., Avila F. (1976). Effect of tRNA from 5-azacytidine-treated hamster fibrosacoma cells on protein synthesis in vitro in a cell-free system. Biochem. Pharmacol..

[24] Cihak A. (1974). Biological effects of 5-Azacytidine in eukaryotes. Oncology.

[25] Reichman M., Penman S. (1973). The mechanism of inhibition of protein synthesis by 5-Azacytidine in HeLa cells. Biochim. Biophys. Acta.

[26] Schaefer M., Hagemann S., Hanna K., Lyko F. (2009). Azacytidine inhibits RNA methylation at DNMT2 target sites in human cancer cell lines. Cancer Res..

[27] Von Hoff D.D., Slavik M., Muggia F.M. (1976). 5-Azacytidine a new anticancer drug with effectiveness in acute myelogenous leukemia. Ann. Intern. Med..

[28] Peterson B.A., Collins A.J., Vogelzang N.J., Bloomfield C.D. (1982). 5-Azacytidine and renal tublular dysfunction. Blood.

[29] Vermeulen K., Van Bockstaele D.R., Berneman Z.N. (2003). The cell cycle: a review of regulation, deregulation and therapeutic targets in cancer. Cell Prolif..

[30] Tobey R.A. (1972). Effects of cytosine arabinoside, daunomycin, mithromycin, azacytidine, adriamycin, and camptothecin on mammalian cell cycle traverse. Cancer Res..

[31] Chabot G.G., Momparler R.L. (1986). Effects of 5-aza-2′-deoxycytidine on suvival and cell cycle progression of L1210 leukemic cells. Leuk. Res..

[32] Momparler R.L., Samson J., Momparler L.F. (1984). Cell cycle effects and cellular pharmacology of 5-Aza-2′-deoxycytidine. Cancer Chemother. Pharmacol..

[33] Wilson V.L., Jones P.A., Momparler R.L. (1983). Inhibition of DNA methylation in L1210 leukemic cells by 5-Aza-2′-deoxycytidine as a possible mechanism of chemotherapeutic action. Cancer Res..

[34] Stresemann C., Lyko F. (2008). Modes of action of the DNA methyltransferase inhibitors azacytidine and decitabine. Int. J. Cancer.

[35] Borthakur G., Ahdab S.E., Ravandi F., Faderl S., Ferrajoli A., Newman B., Issa J.P., Kantarjian H. (2008). Activity of decitabine in patients with myelodysplastic syndrome previously treated with azacitidine. Leukemia Lymphoma.

[36] Raynal N.J., Momparler L.F., Rivard G.E., Momparler R.L. (2011). 3-Deazauridine enhances the antileukemic action of 5-aza-2′-deoxycytidine and targets drug-resistance due to deficiency in deoxycytidine kinase. Leuk. Res..

[37] Hagemann S., Heil O., Lyko F., Brueckner B. (2011). Azacytidine and decitabine induce gene-specific and non-random DNA demethylation in human cancer cell lines. PLoS One.

